# Heads in the clouds: On the carbon footprint of conference‐seeded publications in the advancement of knowledge

**DOI:** 10.1002/ece3.8201

**Published:** 2021-10-12

**Authors:** Laurent Seuront, Katy R. Nicastro, Gerardo I. Zardi

**Affiliations:** ^1^ Univ. Lille, CNRS, Univ. Littoral Côte d’Opale, UMR 8187 – LOG – Laboratoire d’Océanologie et de Géosciences Lille France; ^2^ Department of Marine Resources and Energy Tokyo University of Marine Science and Technology Minato‐ku Japan; ^3^ Department of Zoology and Entomology Rhodes University Grahamstown South Africa; ^4^ CCMAR–Centro de Ciencias do Mar CIMAR Laboratório Associado Universidade do Algarve Faro Portugal

**Keywords:** carbon footprint, climate change, COVID crisis, scientific meetings, scientific production and productivity, virtual conferences

## Abstract

The carbon footprint of flying overseas to conferences, meetings, and workshops to share and build knowledge has been increasingly questioned over the last two decades, especially in environmental and climate sciences, due to the related colossal carbon emissions. Here, we infer the value of scientific meetings through the number of publications produced either directly or indirectly after attending a scientific conference, symposium, or workshop (i.e., the conference‐related production) and the number of publications produced per meeting (i.e., the conference‐related productivity) as proxies for the academic value of these meetings, and relate them to both the number of meetings attended and the related carbon emissions. We show that conference‐related production and productivity, respectively, increase and decay with the number of meetings attended, and noticeably that the less productive people exhibit the largest carbon footprint. Taken together, our results imply that a twofold decrease in the carbon footprint $F_{{\mathrm{CO}}_{2}}$ of a given scientist would result in a twofold increase in productivity through a fivefold decrease in the number of meeting attended. In light of these figures, we call for both the implementation of objective and quantitative criteria related to the optimum number of conferences to attend in an effort to maximize scientific productivity while minimizing the related carbon footprint, and the development of a rationale to minimize the carbon emission related to scientific activities.

## Graphical abstract

We assessed the carbon footprint of the conference‐seeded scientific productivity as the number of publications produced per meeting as a proxy for the academic value of these meetings, and relate them to both the number of meetings attended and the related carbon emissions. We show that scientific productivity respectively decays with the number of meetings attended, and noticeably that the less productive people exhibit the largest carbon footprint.

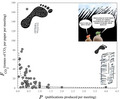

The United Nations Climate Change conferences and the Intergovernmental Panel on Climate Change (IPCC) reports unequivocally indicate that despite the considerable amount of effort related to decrease our carbon footprints through the development of alternative energy solutions and more incentive toward carbon neutral events such as the One Planet Summit, we are still living in an era of increasing anthropogenic pressure on our environment. Ironically, even joining carbon neutral events comes at a considerable cost in terms of traveling carbon footprint. Beyond the undisputed industrial contribution to climate change, our role as scientists to sharing and building scientific knowledge through attending conferences results in a considerable amount of carbon emission (Klöwer et al., 2020).

As scientists, we are travelers. We travel to visit collaborators, to give seminars and lectures, to conduct fieldwork, and many (if not most) of us travel, often using planes, to attend conferences throughout the world, sometimes several times per year. The colossal environmental impacts of traveling to and from these meetings have widely been acknowledged over nearly two decades to account for the largest share of conference‐related carbon emissions (Achten et al., 2013; Bossdorf et al., 2010; Hischier & Hilty, 2002; Stroud & Feeley, 2015) and specifically discussed in medicine (Roberts & Godlee, 2007), psychiatry and neuroscience (Young, 2009), biomedical science (Dwyer, 2013), agriculture (Desiere, 2016), and geography (Nevins, 2014). As an example, the 28,000 delegates traveling to the Fall Meeting of the American Geophysical Union (AGU) held yearly in San Francisco emitted ca. 80,000 tonnes of CO_2_ (Klöwer et al., 2020), the equivalent of the weekly emissions of averaged sized cities such as Edinburgh or Brazilia (Moran et al., 2018). However, the paradox of an ever‐increasing academic travel behavior in an era of global change with urging economic, political, and societal issues including “sustainable mobility” and “active and responsible citizenship” noticeably persists (Caset et al., 2018).

The carbon footprint of flying overseas to conferences, meetings, and workshops to share and build scientific knowledge has repeatedly been discussed, especially in environmental and climate sciences (Fox et al., 2009; Grémillet, 2008; Orsi, 2012; Stroud & Feeley, 2015). Climate scientists even argued that their large carbon footprints decrease their credibility to the public and the impact of their advice (Attari et al., 2016); both the number of and attendance to international forums such as IPCC and Conference of the Parties (COP) meetings are quintessential examples of this paradox. Though some organizations such as the International Coral Reef Society already have a history of “virtual‐only” meetings, virtual conferences blossomed across the world as a response to the COVID‐19 pandemic, with an undisputable success in terms of attendance. As a noticeable example, the 2020 virtual General Assembly of the European Geophysical Union (EGU) gathered 22,376 scientists from 134 countries, a staggering figure when compared to 2019, 2018, and 2017 General Assembly which respectively gathered *only* 16,273, 15,075, and 14,496 scientists from 113, 106 and 107 countries. These observations are consistent with the emerging idea that virtual meetings may represent the future of scientific meetings (Abbott, 2020; Margolis et al., 2020). The 37.5% increase in the attendance to General Assembly of the European Geophysical Union meeting between 2019 and 2020 is unfortunately most likely related to the COVID crisis. The above‐mentioned increase nevertheless indicates that virtual conferences are likely to attract people who may not afford traveling (this is a critical issue for both students and scientists from low‐income countries; this hypothesis is consistent with the 15.7% increase in the number of countries of origin of the attendees, a trend also visible in 2021 with 136 countries represented) or might find difficult to arrange travel such as parents. Note that this example is not an isolated one as, for example, 10% more people attended the virtual European Biological Rhythms Society (EBRS) than physically traveled to the previous conference (Abbott, 2020).

In this context, we considered the number of peer‐reviewed publications produced either directly or indirectly after attending a scientific meeting (i.e., conference, symposium, or workshop; referred to as *conference*‐*related production* hereafter), and the subsequent conference‐related productivity (i.e., number of publications produced per meeting) as proxies for the academic value of these meetings, and we further assessed their carbon footprint. Our approach does not intend to assess productivity as a temporally derived measure (i.e., number of publications produced per unit of time). Instead, scientific productivity, expressed here as the amount of output (i.e., the number of publications) per unit of inputs (i.e., the number of conferences), is sensu stricto a measure of the efficiency of production. We acknowledge that the power of face‐to‐face contact in generating new thinking, ideas, networks, and collaborations—and even in some instances improving the odds of getting a job and receiving grant funding, though evidence exists that academic air travel is of limited influence on professional success (Wynes et al., 2019)—is invaluable aspects of scientific meetings that cannot be underestimated. We nevertheless stress that though the individual benefits of all these activities to *facilitate* thinking and ideas are non‐questionable, they are extremely difficult to assess objectively and quantitatively. Instead, given that the end product of these activities is to *produce* tangible science, we considered conference‐related production and productivity as one of the most straightforward metrics of how scientific meetings contribute to build and spread knowledge, hence to evaluate the scientific value of meetings in aggregate. Note that our approach is not an attempt to measure productivity *per se*, which has been thoroughly studied elsewhere; see, for example, Bradshaw and Brooks (2016) and references therein.

We designed a 7‐question survey to assess (i) how many scientific meetings (workshops, conferences, symposiums) were attended in the last 5 years, (ii) how many of these meetings involved air travel, (iii) the countries of departure and destination for each meeting involving air travel, (iv) how many publications are the direct or indirect result of these meetings (inspired by oral presentations, posters or discussions with colleagues), (v) the willingness to participate to scientific meeting remotely (e.g., via video conferencing), (vi) the reasons for using air travel, and (vii) the willingness to consider eco‐friendly alternative travel options, even if they imply spending more time on the road. The answers were further classified as a function of gender and academic position.

The questionnaire was sent between January and December 2019 to a panel of 211 marine biologists and oceanographers (91 females and 120 males) scattered over 27 countries. Note that we exclusively targeted people working in academia as publications is fundamentally an academic metric. Government, non‐governmental organization, and charity workers who also attend scientific conferences do not have the same motivation to publish than academics, hence have been deliberately omitted from our survey to avoid introducing a bias in the interpretation of our results. We received 76 answers, a response rate of 36%, similarly segregated between females (36.1%) and males (35.8%). These response rates are highly satisfactory for an online survey (Baruch & Holtom, 2008). A total of 709 conferences were attended by these scientists over the period 2015–2019. The number of meetings attended per individual (0–40) was highly significantly positively skewed (*p* < .001) and did not significantly differ between females and males (*p* > .05). Air travel was the preferred means of transportation to attend conferences by, respectively, 64.5% and 78.9% of female and male scientists. A noticeable proportion of them (35.5 and 21.1% of females and males) *intentionally* chose eco‐friendly travel options. We subsequently estimated the carbon footprint of these air travels using the Carbon Fooprint^TM^ calculator by including the DEFRA's recommended Radiative Forcing correction factor of 1.891 (DEFRA, 2016). The overall resulting carbon footprint (471.5 tonnes of CO_2_) corresponds to individual contributions ranging between 0.02 and 14.19 tonnes of CO_2_ per individual per year, in agreement with previous estimates (Klöwer et al., 2020; Spinellis & Louridas, 2013).

The majority of the respondents (84%) published papers that they considered as the direct or indirect results of the meetings they attended over the last 5 years. The number of conference‐related publications *n* produced by each scientist (between 1 and 21) was highly significantly positively correlated to the number of meeting *N* they attended (*r* = .61, *p* < .01; Figure 1a). A significant correlation was found between the carbon footprint and the numbers of meetings (*r* = .65, *p* < .01). The carbon footprint was, however, noticeably not significantly correlated (*r* = .10, *p* > .05) with the number of papers produced. This somehow counter‐intuitive result is due to both the proportion of the respondents (16%) who did not published any papers related to the conferences they attended—though these conferences (2–12) still contributed to their carbon footprint—and the 10‐ to 20‐fold difference in the number of papers produced by people who went to a similar number of meetings (Figure 1a). The number of publications produced per meeting, that is, the conference‐related productivity *P* (*P* = *n*/*N*), decayed as a power‐law function of the number of meetings attended *N* as *P* = 1.164*N* ^−0.454^ (*r* = .40, *p* < .01; Figure 1b). This result indicates a drastic decrease in scientific productivity (i.e., the efficiency of scientific production) with the number of meetings attended. These figures convert into a significant power‐law decay (*r* = .54, *p* < .01) of the carbon footprint $F_{{\mathrm{CO}}_{2}}$ (tonnes of CO_2_ per paper per meeting) as a function of conference‐related productivity *P*, that is, $F_{{\mathrm{CO}}_{2}}$ = 4.535*P* ^−0.934^ (Figure 1c). None of these relationships significantly differ between female and male scientists (*p* > .05). Male scientists noticeably included both the most productive and the greatest carbon emitter. The best performer was among the most carbon friendly scientists, by producing 4 papers per meeting at a cost of 2.2 tonnes of CO_2_. In contrast, the utmost carbon emitter only produced 0.4 paper per meeting worth a staggering 166.9 tonnes of CO_2_, about 4 times the carbon footprint of an inhabitant of an average size city‐like Edinburgh over a period of 5 years. The observed power laws imply that a twofold decrease in the carbon footprint $F_{{\mathrm{CO}}_{2}}$ of a given scientist would result in a twofold increase in productivity through a fivefold decrease in the number of meeting attended. These results quantitatively generalized previous suggestions to decarbonize conference travel by attending fewer scientific conferences (Philippe, 2008), optimizing conference locations (Stroud & Feeley, 2015), and boosting virtual attendance and regional hubs (Klöwer et al., 2020). We acknowledge that the lack of face‐to‐face accountability is one of the biggest concerns with virtual meetings, especially where meetings are audio‐only (Nili & Shaner, 2020), though recent evidence exists that fluent discussions can also be achieved through interactive hubs on social media such as Twitter (Abbott, 2020). The 37.5% increase in the attendance to the virtual EGU General Assembly further stresses the importance of the understated benefits of virtual conferences which, beyond cutting carbon emission, are likely to attract people who may not afford traveling (this is an acknowledged critical issue for students and scientists from low‐income countries; Ćuk et al., 2020) or might find difficult to arrange travel such as parents (Abbott, 2020). Taken together, our results—and in particular the relationship describing the power‐law decay of carbon footprint with productivity, $F_{{\mathrm{CO}}_{2}}$ = 4.535*P* ^−0.934^—may be considered as a first step toward the development of objective and quantitative criteria related to the optimum number of conferences to attend in an effort to maximize productivity while minimizing the related carbon footprint.

**FIGURE 1:**
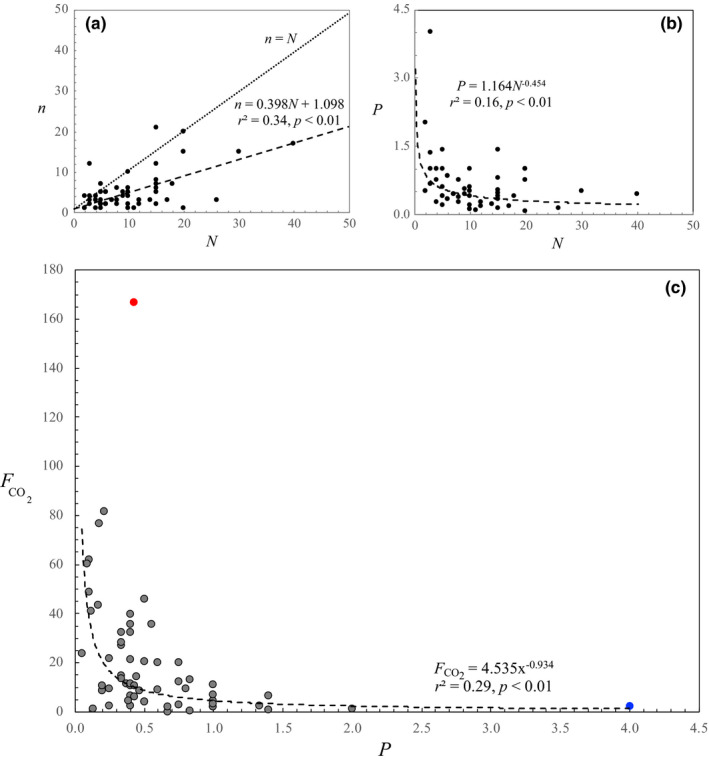
Assessment of the academic value of scientific meetings in building knowledge through (a) the number of scientific publications *n* (produced either directly or indirectly after attending a scientific conference, symposium, or workshop) as a function of the number of scientific meetings attended over a period of 5 years (2015–2019), (b) the scientific productivity *P* (proxied by the number of publications produced per meeting) as a function of the number of meetings attended, and (c) the carbon footprint $F_{{\mathrm{CO}}_{2}}$ (tonnes of CO_2_ per paper per meeting) as a function of the scientific productivity (*P*), expressed as the number of publications produced per meeting over a period of 5 years, where the best performer and the utmost carbon emitter are shown in blue and red, respectively

A vast majority (>90%) of the respondents to our survey was willing to consider eco‐friendly alternative travel options (Figure 2a), though discrepancies exist between males and females, especially with regard to their academic positions (Figure 2b,c). Noticeably, males with higher positions exhibit a decreasing willingness to consider eco‐friendly alternative travel options, a trend that is non‐existent for females (Figure 2c,d). Over 60% of the respondents would participate to meetings remotely (Figure 2d), acknowledged that virtual meeting would lose some value, but considered the trade‐offs as acceptable given the environmental benefits, irrespective of their academic positions (Figure 2e,f).

**FIGURE 2:**
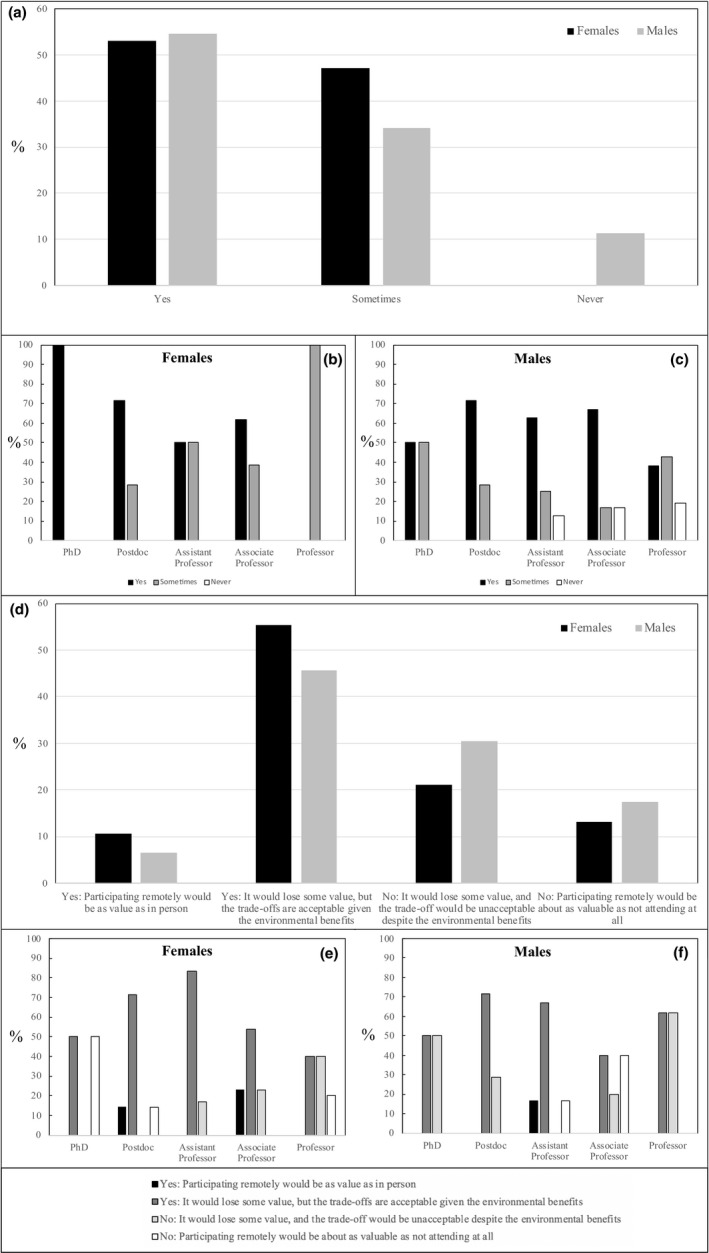
Willingness of both female and male scientists (a) to consider eco‐friendly alternative travel options, even if they imply more time on the road, and (d) to participate to scientific meetings remotely (e.g., via video conferencing or other technologies). The former and the latter questions have further been considered for females (b, e) and males (c, f) depending on their career stage

Some respondents (13.2%) shared extra comments and thoughts. The majority of them were positive, acknowledging the timely nature of the issue, and converged toward the facts that we, as a scientific community, are “*very big offenders*,” and even though “*the efficiency of remote meetings has long been hampered by technological limitations especially in some countries*” where “*internet connections are not always ideal*,” there was a consensus that “*alternative solutions are needed*” and should be facilitated by the fast‐improving technology. Two respondents were, however, hostile to the scope of the study. The first one only stated “*If you intend to use the results of this study in any format to make comment on the value of conferences then you may be doing the world of research a disservice*.” The second one first stating “*I don't see the point of such an initiative*,” further argued that “*we produce because we work! Going at sea and flying to conferences is work. If we have to produce less, let's quit our jobs and close our labs*.” Though the former is at best counter‐productive, the latter touches a seemingly soft spot in numerous scientists who are backed up by the misconception that “we travel hence we produce” rather than “we produce and we limit to essential traveling,” which is possibly one of the very few silver linings of the COVID crisis.

Although the current study is based on a relatively limited sample, it indicates that scientific productivity decays with the number of meetings attended and that the less productive people exhibit the largest carbon footprint. Though conducted in a different context, these results are consistent with broader discussions about the detrimental effect meetings may have on productivity (Perlow et al., 2017; Rogelberg et al., 2007). In the light of the COVID crisis and the resultant blossoming of virtual conferences, our results further question the relevance of attending scientific meetings physically, which generate exorbitant carbon footprints, as a mean of knowledge creation. This issue is particularly timely as, beyond the specific issue of conference attendance, a relatively recent message (October 21, 2020) released by the Headquarters of the Centre National de la Recherche Scientifique (CNRS) urges French scientists to rethink their professional practices to decrease, and eventually minimize, their carbon footprints. Beyond the discussions about whether meetings have scientific value in aggregate, this initiative stresses the need to adjust expectations in light of their climate costs, reduce the carbon footprint of those activities, or internalize the carbon costs. We further stress that our survey exclusively targeted academic people as scientific production and productivity is fundamentally an academic metric. However, government, non‐governmental organization, and charity workers who also attend scientific conferences do not have the same motivation to publish than academics. As such, they have been deliberately omitted from our survey to avoid introducing a bias in the interpretation of our results. Though the resolution of this specific issue lies far beyond the scope of the present work, the assessment of the benefits of their conference‐related carbon footprint warrants the need for further work.

At the individual level, cutting down CO_2_ emissions related to scientific meetings would necessitate avoiding traveling to unessential meetings and/or prioritizing events with small carbon footprints. Under the assumption that people respond to incentives and act in their own interest (e.g., productivity, but also job hunting, employer expectations, and socialization) in choosing to attend meetings—and more meetings occur to supply that demand—our results show that attending more meeting may actually be counter‐productive in terms of efficiency of production, specifically proxied here by the number of publications published per meeting. In this context, it is worth noting that our approach is independent of the nature of one's publication record. Scientists with stellar publication records—who often attend a lot of conferences and meetings as invited and/or plenary speakers—are unlikely to turn their conference‐related carbon footprint into publications, but rather benefit the audience from their experience. The subsequent “return on carbon investment” is difficult to quantify, though it can easily be optimized through a virtual model. In turn, at the collective level, and given the non‐negligible function of scientific conferences to generate income for universities and scientific societies—not to mention the profitable industry of conference organization—there is also a critical need in defining a new sustainable economic model to operate much needed low‐carbon footprint scientific conferences through, for example, relocating carbon expansive events, decreasing the frequency of meetings, increasing virtual participation, or eventually moving to a virtual‐only model (Klöwer et al., 2020).

Despite the scientists' recognition of the realities of climate change, the international nature of their activities and the need to maintain their societal credibility by leading by example in reducing their carbon footprint, our results suggest that the implementation of a low‐carbon research culture for the 21th century advocated by the Tyndall Centre for Climate Change Research (Le Quéré et al., 2015) may still be in its infancy.

## CONFLICT OF INTEREST

The authors have no conflicts of interest.

## AUTHOR CONTRIBUTIONS

**Laurent Seuront:** Conceptualization (equal); investigation (equal); methodology (equal); formal analysis (lead); writing—original draft (lead); and writing—review & editing (lead). **Katy R. Nicastro:** Conceptualization (equal); investigation (equal); methodology (equal); formal analysis (equal); and writing—review & editing (equal). **Gerardo I. Zardi:** Conceptualization (equal); investigation (equal); methodology (equal); formal analysis (equal); and writing—review & editing (equal).

## Data Availability

The original version of our questionnaire as well as the collected answers is freely available in the Dryad data repository (https://doi.org/10.5061/dryad.8sf7m0cp9).
